# Reproductive Trade-Offs May Moderate the Impact of *Gyrodactylus salaris* in Warmer Climates

**DOI:** 10.1371/journal.pone.0078909

**Published:** 2013-10-30

**Authors:** Scott J. Denholm, Rachel A. Norman, Andrew S. Hoyle, Andrew P. Shinn, Nick G. H. Taylor

**Affiliations:** 1 Computing Science and Mathematics, School of Natural Sciences, University of Stirling, Stirling, United Kingdom; 2 Institute of Aquaculture, School of Natural Sciences, University of Stirling, Stirling, United Kingdom; 3 Centre for Environment, Fisheries and Aquaculture Science (Cefas), Weymouth Laboratory, Weymouth, United Kingdom; McGill University, Canada

## Abstract

*Gyrodactylus salaris* is a notifiable freshwater ectoparasite of salmonids. Its primary host is Atlantic salmon (*Salmo salar*), upon which infections can cause death, and have led to massive declines in salmon numbers in Norway, where the parasite is widespread. Different strains of *S. salar* vary in their susceptibility, with Atlantic strains (such as those found in Norway) exhibiting no resistance to the parasite, and Baltic strains demonstrating an innate resistance sufficient to regulate parasite numbers on the host causing it to either die out or persist at a low level. In this study, Leslie matrix and compartmental models were used to generate data that demonstrated the population growth of *G. salaris* on an individual host is dependent on the total number of offspring per parasite, its longevity and the timing of its births. The data demonstrated that the key factor determining the rate of *G. salaris* population growth is the time at which the parasite first gives birth, with rapid birth rate giving rise to large population size. Furthermore, it was shown that though the parasite can give birth up to four times, only two births are required for the population to persist as long as the first birth occurs before a parasite is three days old. As temperature is known to influence the timing of the parasite's first birth, greater impact may be predicted if introduced to countries with warmer climates than Norway, such as the UK and Ireland which are currently recognised to be free of *G. salaris*. However, the outputs from the models developed in this study suggest that temperature induced trade-offs between the total number of offspring the parasite gives birth to and the first birth timing may prevent increased population growth rates over those observed in Norway.

## Introduction

Globally, wild Atlantic salmon, *Salmo salar* L., are in decline and their conservation is of great concern [1]. In particular, Norway has experienced massive declines in its Atlantic salmon populations, which have largely been attributed to gyrodactylosis. This disease, which predominantly affects juvenile stages of Atlantic salmon in freshwater, is caused by the viviparous ectoparasite *Gyrodactylus salaris* Malmberg, 1957 [2]–[5]. Since its first report in Norway in 1975, *G. salaris* has since been reported from 46 rivers [6]. It is estimated that *G. salaris* has reduced the average density of salmon parr in infected rivers by up to 86% [7], and costs the Norwegian economy over US $50 M p.a. through the costs of surveillance and eradication (circa US $23 M p.a.), and losses to fisheries associated industries and tourism (circa US $34 M p.a.) [6]. As a consequence of its impact, countries recognised by the European Commission (EC) as free of *G. salaris*, such as the United Kingdom (UK) and Ireland (Commission decision 2004/453/EEC), are keen to determine whether the parasite could establish under their environmental conditions if introduced, and if so, the impact it may have.

Strains of Atlantic salmon native to Norway and the UK are highly susceptible to *G. salaris* infections [8]–[10] and on juvenile hosts the parasite population is able to increase exponentially and cause substantial mortality [11], [12]. The parasite generally has short generation times with adults giving birth to fully grown pregnant offspring (hyperviviparity). This polyembryonous, progenetic method of reproduction allows the parasite population to grow rapidly to epidemic levels within a susceptible host population. Baltic strains of Atlantic salmon, however, seem capable of regulating the parasite population and are able to coexist with the parasite due to some form of innate resistance [13]. Studies on this resistance show that Baltic strains of salmon up-regulate INFγ, Mx and MHC I genes post-infection and reduce mucous cell density in response to infection, whereas Atlantic strains do not [14]. Experimental evidence suggest that in Baltic strains of salmon host responses are able to decrease the number of offspring *G. salaris* are able to produce, reduce their survival and delay the rate at which the parasite gives birth [15]. The potential for parasite population regulation due to host immunity is also supported by modelling studies on other gyrodactylid / host systems [16]–[19], though these do not demonstrate which life history traits are influential in driving regulation, and are assumed to be representative of the *G. salaris* / Atlantic salmon system.

Though the UK and Ireland have recognised freedom from *G. salaris*, the conservation of wild Atlantic salmon is a key concern of their respective governments and there is great anxiety over the potential impact should the parasite be introduced. Consequently a large body of research has been undertaken to determine the routes by which the parasite may be introduced and develop contingency plans to prevent introduction and control spread if required [20]. More recent modelling work has been carried out by Ramírez *et al.* [21] with the aim of estimating the error in gyrodactylid population growth rates subject to stochastic variation in survivorship and reproduction by the means of an individual agent-based model of *G. salaris* infection on a single salmon host., Little research has, however, been conducted to establish the likely impact of the parasite if introduced to the UK or Ireland. Though UK salmon strains are known to be susceptible to the parasite, environmental conditions, especially temperature and seasonality are very different to those of Norway. Differences in such environmental conditions are known to alter the timings of the parasite's life-cycle and may, therefore, influence population success and size [22]–[23]. Identifying and understanding the key life-history traits that are most influential in shaping the resulting population dynamics of the parasite is one of the first steps in allowing more informed predictions of impact in the event of an introduction. The present study aimed to investigate this by modelling the parasite's life-cycle to determine the sensitivity of *G. salaris* population dynamics on a single salmon host to changes in key life-stage timings and events, such as survival and reproduction rates, and the total number of offspring produced.

## Methods

In order to allow cross validation of the study results, two independent modelling approaches were taken. The first was a Leslie matrix model, which provides a widely used discrete time age/stage structured approach to modelling population growth [24]. The second was a continuous time compartment based model where parasites were assumed to pass through a series of states as they developed. In both instances deterministic models were first built in order to assess model fit to experimental data and the sensitivity of population growth to manipulation of stage level and overall rates. Both models were also adapted to include stochasticity in order to understand the range of behaviours, *i.e.*, outbreak sizes that were likely to occur, and compare changes in extinction probabilities.

### Approach 1 – Leslie matrix model

A 26×26 Leslie matrix model was developed to predict the growth of a *G. salaris* population over a 30 day period. This was based on *G. salaris* having a maximum longevity of 26 days on either salmon strain (figures derived from [15]). The model was developed so that each *G. salaris* parasite gave birth to either four or two offspring in its lifetime depending on whether it was infecting a susceptible or resistant Atlantic salmon strain respectively (Table 1). The days on which offspring were born were used to calculate the proportion of parasites giving birth on each day of their 26 day life-span. The Leslie matrix itself is a square (ω×ω) matrix (the transition matrix) that is closed to migration (Equation 1). The matrix only considers the female population, but in the case of *Gyrodactylus*, all individuals can be considered female.

**Table 1:** Life history parameter values for *Gyrodactylus salaris* infecting resistant or susceptible Atlantic salmon strains derived from Cable *et al*. (2000).

Parameter	Resistant (Neva) host	Susceptible (Alta) host
**Model 1**		
Daily survival rate (*P_i_*):	0.82	0.92
Max survival time (days):	17 (P_17_ = 0)	24 (P_24_ = 0)
Daily birth probabilities (*F_i_*):		
*F_1_*	0.00	0.15
*F_2_*	0.66	0.85
*F_3_*	0.34	0.00
*F_9_*	0.00	0.95
*F_10_*	1.00	0.05
*F_16_*	0.00	0.60
*F_17_*	0.00	0.40
*F_22_*	0.00	0.50
*F_23_*	0.00	0.50
**Model 2**		
Daily mortality rates (μ):		
μ_V_	1/2.75	1/6.00
μ_F_	1/2.90	1/4.50
μ_S_	1/2.00	1/5.00
μ_T_	0.00	1/4.00
μ_E_	0.00	1/5.00
Daily birth rates (λ):		
λ_v_	1/2.34	1/1.85
λ_F_	1/7.66	1/7.20
λ_S_	0.00	1/7.30
λ_T_	0.00	1/6.00
**Total births:**	2	4

Subscript *i*  =  age (in days from 1 to 26). Values of *F_i_* not listed  =  0. Subscripts: V, F, S, T, E relate to model 2 stages. Model 1 refers to the Leslie matrix model and model 2 refers to the compartmental model.

All elements in the Leslie matrix are zero except those found in the first row, which correspond to fertility rates, F_i_>0, (elements of the form a_1,j_, j = 1, …, ω) and sub-diagonal, which correspond to the survival rates, 0<P_i_<1 (elements of the form a_i+1,i_, i = 1, …, ω-1). Finally, the number of individuals in each age-class at time t+1 is derived when the transition matrix is multiplied by a column vector n_i_(t) that contains the number of individuals in each age class i at time t.
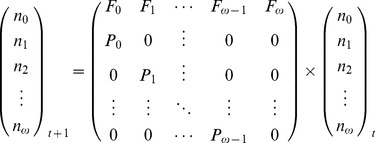
(Eq 1)


The overall rate of population growth was determined by calculating the dominant eigenvalue (*λ*) for the transition matrix given a set of parameter values. If *λ*<1 then the population declines over time, if *λ* = 1 the population remains constant, and if *λ*>1 the population grows exponentially. This value was used to assess the influence of altering rates within the matrix on the resulting population dynamics.

In addition to the Leslie matrix model, an individual based stochastic model was also developed. As with the Leslie matrix model, this consisted of 26 stages representing the 26-day longevity of individual parasites. At each time step, each parasite could either die or progress to the next stage. To determine whether a parasite progressed to the next stage or died, a random number (between 0 and 1) was generated and compared to the survival probability. If this value was less than the survival rate, then the parasite died, otherwise it moved to the next stage. At certain stages (*i.e.* at days 1.85, 9.05, 16.40 and 22.5), the parasite would do one of four things: 1) give birth to an offspring before moving to the next stage; 2) give birth to an offspring before dying; 3) move to the next stage without giving birth; and, 4) die before giving birth. To determine if and when a parasite gives birth, a second random number was generated and compared to the probability of a parasite giving birth on that day. New born parasites entered the model at stage 1. The model followed individual parasites through time and kept track of the number of parasites in each stage throughout the simulation.

### Approach 2 – Compartment based model

**Figure 1:**
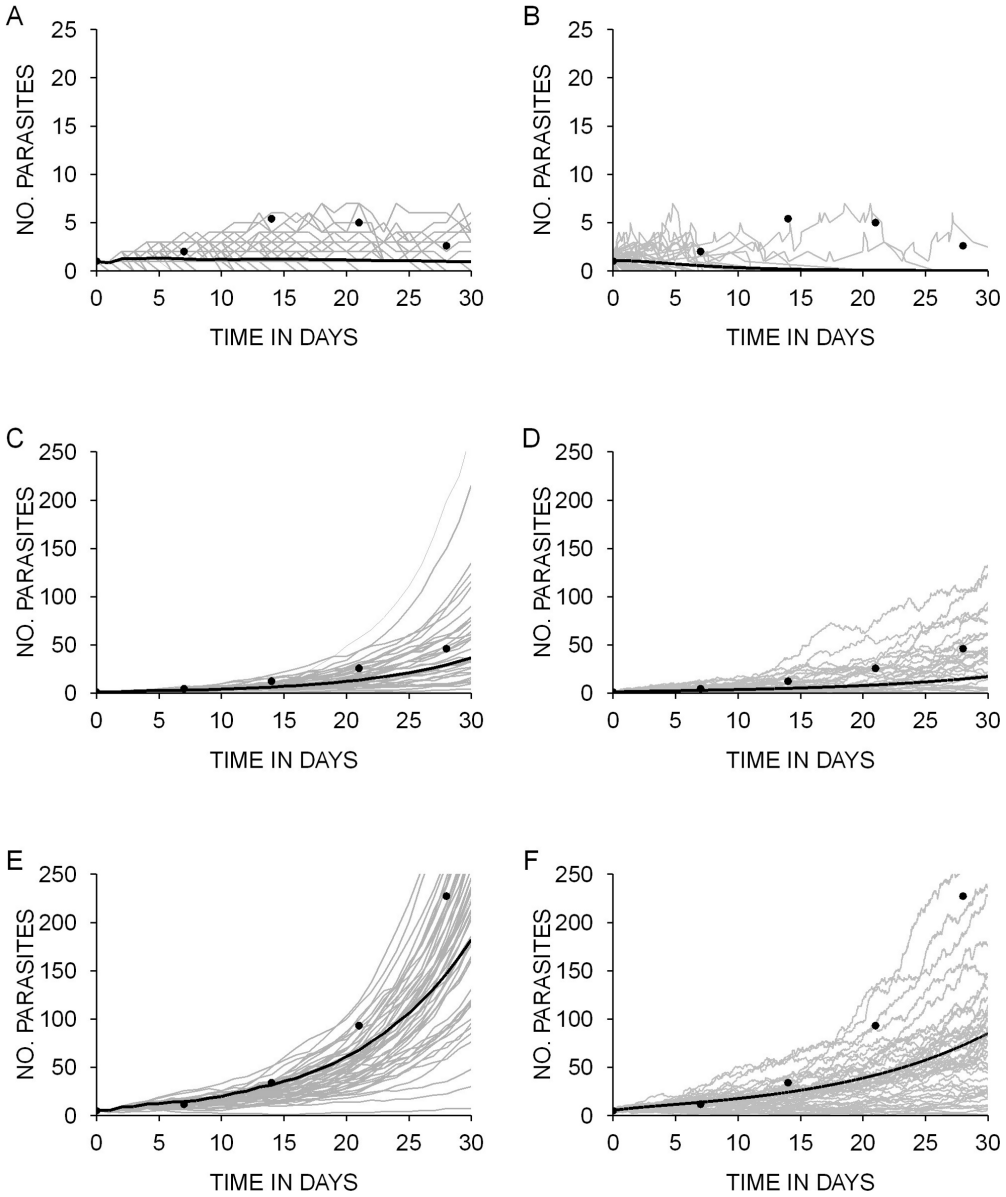
Dynamics of *Gyrodactylus salaris* infecting individual Atlantic salmon hosts. Black lines  =  deterministic model predictions, grey  =  stochastic simulation runs, black points  =  published experimental data [26]. Top (a, b)  =  resistant Atlantic salmon strain, starting with one parasite, a  =  Leslie matrix model (model 1), b  =  compartmental model (model 2). Middle (c, d)  =  susceptible Atlantic salmon strain, starting with one parasite, c  =  model 1, d  =  model 2. Bottom (e, f)  =  susceptible Atlantic salmon strain, starting with five parasites, e  =  model 1, f  =  model 2.

To ensure consistency and add confidence in the accuracy of findings of the above approaches, a compartment based model described by a series of coupled ordinary differential equations (Eq 2 to 6) was derived to provide a deterministic continuous time alternative. This model allowed the population to move through a series of five states from new born (V), to parasites that had given birth once (F), twice (S), three (T) or four (E) times (Figure 1). In each state, parasites gave birth at a rate specific to that state, defined by *λ* and the appropriate subscript for that state (*i.e.* V to E). A state specific mortality rate was also included defined by *μ* and the appropriate subscript for that state. A maximum of four births was assumed [15], but to reduce this, *λ* was set to 0 for states occurring after the desired number of births had been achieved. 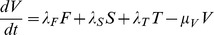
(Eq 2)

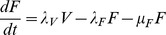
(Eq 3)

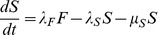
(Eq 4)

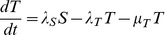
(Eq 5)

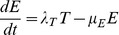
(Eq 6)


The intrinsic population growth rate (r) for this model was calculated using equation (7) where r is the total number of offspring an average parasite will produce in its lifetime. At each stage of a parasite's life-cycle (V, F, S and T classes as above) a parasite can either reproduce or die. Thus, given that only one offspring will be produced by an adult parasite, the number of offspring produced at each stage by an “average” parasite is calculated. Adding together the average number of offspring produced at each stage we arrive at r in equation (7). In this case when r<1 the population will decline, when r = 1 the population will remain constant, and when r>1 the population will grow exponentially. This was used to assess the influence of parameter changes on population growth.
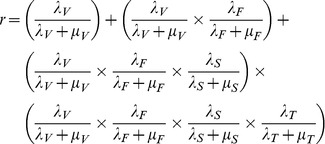
(Eq 7)


In order to determine the influence of random processes on the dynamics and population success of the parasite, this model was also programmed to incorporate demographic stochasticity using the methods proposed by [25].

### Parameterisation, simulation and sensitivity analysis

Models were initially parameterised assuming daily rates based on data from [15] for *G. salaris* infecting either a resistant (River Neva) or susceptible (River Alta) salmon strain (Table 1). Both models assumed that resistant salmon consistently provided a less suitable environment for the parasite and the host immune response was not modelled explicitly. Deterministic and stochastic model predictions were compared to each other and to data from [26] in order to assess the accuracy each modelling approach in capturing the population dynamics of the parasite on susceptible and resistant host strains. Data from [15], [26] was based on rates observed at water temperatures of 12°C to 13°C. In accordance with [26], simulations were initiated with one parasite assumed to be in the earliest life stage and run for 30 days.

The parameter values used for the resistant host strain were taken as a baseline on which to conduct sensitivity analysis. This involved either reducing the total number of births, or reducing reproductive or survival rates by one day for each life stage, or across all stages. The effect of such changes on the growth rate and final population size (in the case of the deterministic models), and the extinction probability based on 1000 simulations (in the case of the stochastic models) was then monitored.

## Results

Published data [26] pertaining to the population dynamics of *G. salaris* on resistant Baltic strains of Atlantic salmon when infected with one parasite show an initial increase in numbers before a decline after day 15 post-infection (Figure 1a, 1b). Neither deterministic model outputs demonstrated this dynamic, with the model 1 output staying fairly constant at around one parasite per fish for the 28 day period studied (Figure 1a), and the model 2 population going extinct by day 17 post-infection (Figure 1b). For both modelling approaches the rates of population increase (*λ* and r) predicted for *G. salaris* on resistant Baltic Atlantic salmon strains were less than one (*λ* = 0.98 and r = 0.69) (Table 2). Some of the stochastic model runs from model 1 appear to provide a similar dynamic to that reported by [22], but in 81% of simulations the population died out before day 30 (Table 2). None of the stochastic model 2 simulations demonstrated a similar dynamic to the reported dynamics and in 98% of cases the parasite population became extinct (Table 2).

**Table 2:** Sensitivity of *Gyrodactylus salaris* population to changes in key parameters within each modelling approach assuming starting N = 1.

	Model 1			Model 2			
**Change to Atlantic salmon baseline**	***λ***	**N at t = 30**	**Extinction Probability**	**R**	**N at t = 30**	**Extinction Probability**	
Baseline Atlantic (4 births)	1.12	32.76	0.29	1.23	16.97	0.62	
Baseline Baltic (2 births)	0.98	0.62	0.81	0.69	0.01	0.98	
Final birth removed	1.11 (−0.9)	31.55 (−3.7)	0.31 (+2)	1.18 (−4.1)	12.96 (−23.6)	0.63 (+1)	
Final 2 births removed	1.10 (−1.8)	25.52 (−22.1)	0.34 (+5)	1.06 (−13.8)	4.84 (−71.5)	0.72 (+10)	
Final 3 births removed	0.96 (−14.3)	2.59 (−92.1)	0.47 (+18)	0.76 (−38.2)	0.06 (−99.6)	0.99 (+37)	
Survival reduced by 10%	1.03 (−8.0)	3.48 (−89.4)	0.65 (+36)	1.16 (−5.7)	9.63 (−43.3)	0.67 (+5)	
1^st^ birth delayed by 1 day	1.09 (−2.7)	11.98 (−63.4)	0.37 (+8)	1.09 (−11.4)	3.02 (−82.2)	0.79 (+17)	

Numbers in parentheses refer to the percentage change from the baseline values. Model 1 refers to the Leslie matrix model and model 2 refers to the compartmental model.

On susceptible strains of Atlantic salmon, *G. salaris* numbers were reported by [26] to grow exponentially over a 30 day study period, whether the population was initially seeded with one (Figure 1c, 1d) or five (Figure 1e, 1f) parasites per fish. Using the parameter estimates gained for *G. salaris* development on susceptible Atlantic salmon strains, both models predicted an intrinsic rate of population growth of greater than 1 (*λ* = 1.12 and r = 1.23) (Table 2). Consequently, both approaches captured the reported exponential dynamic, though both under predict the rate of increase under each circumstance (Figure 1c, 1d, 1e, 1f). This underestimate was particularly evident in the case of model 2 when the population was initially seeded with five parasites (but also apparent when initially seeded with one), as it only predicted around a third of the parasites actually reported at day 28. Under this scenario, though the reported data was found to be within the range of the stochastic run predictions, only a few runs exceeded it. In contrast, a far higher number of stochastic runs produced by model 1 gave predictions closer to the reported data when the population was initially seeded with five parasites. Stochastic runs for *G. salaris* on susceptible Atlantic salmon from model 1 were also around half as likely to predict extinction within 30 days than the stochastic runs from model 2, 0.29 compared to 0.62 (Table 2).

In both models, preventing the final three of four births caused the intrinsic rate of population growth to drop below 1, and substantially increasing the probability of the population going extinct by day 30 post-infection. Model 2 was, however, more sensitive to this change than model 1 predicting the population to go extinct in 99% of stochastic runs and the deterministic result being near extinction by this time. This result however is to be expected as by limiting the parasites to one birth, the total population cannot increase (other than by parents and offspring existing simultaneously) as the parasites can only replace themselves through reproduction, until one dies before it can give birth and the population subsequently dies out.

When either the fourth, or third and fourth births were prevented, both models still predicted intrinsic rates of population growth of greater than one. However, under both scenarios, model 2 was again more sensitive to the changes and extinction probabilities were far higher and final predicted numbers were far lower than those predicted by model 1. Model 1 was, however, more sensitive to a reduction in the overall survival rate of the parasite, and though the proportion of runs going extinct by day 30 was similar to that predicted by model 2, the number of parasite predicted by the deterministic model at day 30 was less than half that predicted by model 2. After the effect of preventing the final three births, model 2 was most sensitive to a delay in the time taken to give birth for the first time. Delaying this by 24 h caused the number of parasites predicted at day 30 to drop to around a fifth of the baseline value and led to a large increase in the extinction probability. Model 2 was also sensitive to this delay in first birth, with numbers predicted at day 30 dropping to around a third of the base line accompanied by a moderate increase in the probability of extinction.

In the case of model 2, it was possible to evaluate the sensitivity of the outputs to changes in the mortality and birth rates for each state in the model independently (Table 3). This revealed that in all cases the number of parasites predicted at day 30 was more sensitive to change in a state's birth rate than its survival rate, though little difference was observed in extinction rates. The largest reduction in parasite numbers was observed for changes to the rates for the first parasite stage. The overall survival rate also had a greater influence on parasite numbers that the overall mortality rate. Obviously a reduction in both of these led to a large reduction in predicted parasite numbers, however, this had less influence than was observed for a 24 h delay in the first birth rate (Table 2).

**Table 3:** Sensitivity of *Gyrodactylus salaris* population to changes in key parameters within each population state in model 2.

Parameters altered from susceptible Atlantic salmon baseline values	r	N at t = 30	Extinction Probability
All survival & birth rates reduced 10%	1.09 (11.4)	4.32 (74.5)	0.75 (13)
All birth rates reduced by 10%	1.15 (6.5)	7.60 (55.2)	0.66 (4)
First birth rate reduced by 10%	1.20 (2.4)	10.77 (36.5)	0.64 (2)
First stage survival reduced by 10%	1.20 (2.4)	12.97 (23.6)	0.66 (4)
Second birth rate reduced by 10%	1.20 (2.4)	12.83 (24.4)	0.63 (1)
Second stage survival reduced by 10%	1.20 (2.4)	13.85 (18.4)	0.66 (4)
Third birth rate reduced by 10%	1.22 (0.8)	15.70 (7.5)	0.64 (2)
Third stage survival reduced by 10%	1.22 (0.8)	16.06 (5.4)	0.63 (1)
Fourth birth rate reduced by 10%	1.22 (0.8)	16.66 (1.8)	0.63 (1)
Fourth stage survival reduced by 10%	1.22 (0.8)	16.73 (1.4)	0.64 (1)
Final stage survival reduced by 10%	1.23 (0.0)	16.94 (0.2)	0.63 (1)
Atlantic (baseline values)	1.23	16.97	0.62
Baltic (baseline values)	0.69	0.01	0.98

Numbers in parentheses refer to the percentage decrease from the baseline values. Model 2 refers to the compartmental model.

## Discussion

*Gyrodactylus salaris* is an internationally important ectoparasite of salmonids that has led to declines in Norwegian Atlantic salmon populations [27]–[31]. The modelling approaches taken in this study increase our understanding of the life-cycle characteristics that drive the parasite's dynamics, and allow inferences to be made regarding the influence different environmental conditions are likely to have on the parasite's impact. This information is critical to identifying ‘at risk’ populations on which surveillance efforts should be focussed [32].

Neither of the modelling approaches used in this study were able to accurately capture the dynamics of the parasite on resistant Baltic Atlantic salmon strains. The results suggested that such host strains do not exhibit increased resistance from the point of infection, but that the response is dynamic, taking over 15 days to reduce the rate of parasite population increase. This evidence for a dynamic response is in keeping with experimental evidence by [14] who demonstrated up regulation in MHC I and INFγ immune relevant genes from day 14 post-infection in resistant Atlantic salmon strains.

Both modelling approaches did reflect the exponentially increasing dynamic of the parasite on susceptible Atlantic salmon strains, suggesting an effective immune response is not mounted by the host. Indeed, both models under predict published rates of parasite population increase, which could simply be a reflection of modelling a stochastic processes (for which there is some evidence, as the published data was located within the range of behaviours predicted by the stochastic simulations), or may suggest that as the infection progresses the host actually becomes more susceptible, potentially due to increasing stress levels or reduced energy reserves. Harris *et al.* [33] and Dalgaard *et al.* [9] both provide some evidence for this hypothesis as they demonstrated that the exposure of susceptible Atlantic salmon strains with corticosteroids known to be linked with stress in fish led to a more rapid increase in *G. salaris* numbers compared to control fish. Additionally, Lindenstrøm *et al.* [34] demonstrated that expression of the cytokine IL-1β was increased in the skin of susceptible salmon circa 14 days post-infection with *G. salaris*. This response was linked with an increase in mucous cell production, which the authors hypothesise was beneficial to the parasite, thus suggesting the environment becomes more hospitable to the parasite as the infection progresses.

Of the modelling approaches used, the Leslie matrix model and associated individual based model (IBM) more accurately captured the dynamics of *G. salaris* infecting susceptible Atlantic strains observed by Bakke *et al.* [26]. This may be a consequence of the event ordered nature of this approach, in which all individuals in an age class capable of giving birth die after the birth has occurred. This is in contrast to the continuous time modelling approach in which births and deaths occur either simultaneously in the case of the deterministic model, or in an unordered fashion in the stochastic model. The superior fit of the Leslie matrix approach indicates that the likelihood of mortality increases either during or for a short period after birth. This finding is supported by the observations of Cable *et al.* [15] who found *G. salaris* mortality to increase 2 days post-birth.

Sensitivity analysis conducted on both models suggests that the third and fourth births of the parasite have little influence on the resulting population dynamics. The timing at which the first parasite birth occurred was, however, shown to have a large amount of influence on the resulting dynamic, with early first births leading to rapid population increases. The timing of subsequent births was less important, but in all cases the stage specific birth rate had greater influence on the population than the stage specific mortality rate. These findings are in keeping with the experimental observations of Cable *et al*. [15], who also hypothesise that the first birth timing is critical in determining the resulting population dynamic. Jansen and Bakke [22] demonstrated that temperature has a strong influence on the birth timing of *G. salaris*, and is thus likely to have a large influence on the parasite's population dynamics and therefore impact in any given environment. As temperature increased they found the time until first birth to decrease. This finding implies that there could be increased impact attributed to the parasite under future climate change scenarios or if introduced to countries with warmer climates than experienced in Norway.

According to Weatherbase (www.weatherbase.com), average summer air temperatures in the UK are around 16°C which is 2.5°C warmer than experienced in Norway (mean = 13.5°C) where *G. salaris* causes problems. By re-analysing the work of Jansen and Bakke [22], the relationship between temperature and first birth timing can be described by log(t) = 2.521−0.133*C, where; t = time in days and C = temperature (°C). Based on this relationship, and assuming the difference in air temperatures between the two countries is also reflected in water temperatures, the average first birth timing in summer in Norway would be over half a day slower than in the UK (2.06 days compared to 1.48 days respectively). Based on the models developed in the present study this half day increase could lead to a 1.09 fold increase in the rate at which parasite numbers increased in the UK compared to Norway. However, Jansen and Bakke [22] also found the average number of births to decrease by two at these temperatures, and when this is accounted for a 1.07 fold decrease below the baseline value of the intrinsic rate of population growth is predicted. This value does, however, remain positive, suggesting that the parasite would still become established under these conditions, but population growth would occur at a slower rate in the UK than observed in Norway. This may also suggest that current Norwegian temperatures are around optimum for *G. salaris* population growth, and should the parasite be introduced to the UK, its impact may be lower. This observation is, however, also relevant within Norway, as, if future climate change scenarios are accurate, average Norwegian air temperatures are predicted to rise by 1.5°C and 3.5°C within the next 50 years [35]. Even at the extreme end of this prediction, this increase would only likely result in a reduction in the first birth timing of 0.77 days, which after accounting for a reduction in total births would result in a 1.03 fold reduction compared to the current predicted population growth rates, potentially meaning the parasite's impact may be reduced. These predictions are confirmed by experimental data from Jansen and Bakke [22] that showed similar rates of population growth at both 13°C and 16.5°C. However, as also observed by Jansen and Bakke [22], the models predict that should temperatures rise to 19°C or above, the rate of first birth timing would increase sufficiently to overcome the influence of the reduction in total births, and the population growth rate would be expected to increase above the current baseline.

Life history theory suggests that species that have low survival rates or high rates of juvenile mortality are expected to evolve to reproduce at a younger age, even though this may reduce lifetime fecundity, than congener species with higher rates of survival [36]. *Gyrodactylus salaris*, like all members of the genus, are hyperviviparous which makes them highly successful colonisers. The offspring are fully grown young which contain developing embryos *in utero*, consequentially, each birthing event can be traumatic with high rates of both parent and daughter mortality [15]. Kochin et al.'s [36] discussion of the adaptive mechanisms displayed by parasitic species in new environments suggests that parasites could evolve a compromise life history or a plastic response with the parasite adopting different strategies suited to each new host / environment. The findings from the current study indicate that response exhibited by *G. salaris* is a trade-off between a decreased longevity in favour of a higher birth rate as a compensatory reaction to surmount the new host's immune system.

This study has highlighted how mathematical modelling techniques can be employed in order to investigate salmon-*G. salaris* interactions, and thus, adds to the findings of previous modelling studies [21], [37]–[44]. Further research is required to incorporate the data derived from the present study into host/parasite models to establish how changes to the parasite's population growth rate may affect transmission and the overall impact to host population.
